# Comparative analysis of microRNA expression in mouse and human brown adipose tissue

**DOI:** 10.1186/s12864-015-2045-8

**Published:** 2015-10-19

**Authors:** Isabelle Güller, Sarah McNaughton, Tamsyn Crowley, Vicente Gilsanz, Shingo Kajimura, Matthew Watt, Aaron P. Russell

**Affiliations:** Centre for Physical Activity and Nutrition, School of Exercise and Nutrition Sciences, Deakin University, 221 Burwood highway, Burwood, VIC 3125 Australia; School of Medicine, Deakin University, Waurn Ponds, Australia; Australian Animal Health Laboratory, CSIRO Animal, Food and Health Sciences, Geelong, Victoria Australia; Department of Radiology, Childrens Hospital Los Angeles, University of Southern California, Los Angeles, California USA; UCSF Diabetes Center, Department of Cell and Tissue Biology, University of California, San Francisco, USA; Department of Physiology, Monash University, Clayton, Victoria 3800 Australia

**Keywords:** microRNA, Brown adipose tissue, Skeletal muscle, Obesity, Type 2 diabetes

## Abstract

**Background:**

In small mammals brown adipose tissue (BAT) plays a predominant role in regulating energy expenditure (EE) via adaptive thermogenesis. New-born babies require BAT to control their body temperature, however its relevance in adults has been questioned. Active BAT has recently been observed in adult humans, albeit in much lower relative quantities than small mammals. Comparing and contrasting the molecular mechanisms controlling BAT growth and development in mice and humans will increase our understanding or how human BAT is developed and may identify potential therapeutic targets to increase EE. MicroRNAs are molecular mechanisms involved in mouse BAT development however, little is known about the miRNA profile in human BAT. The aims of this study were to establish a mouse BAT-enriched miRNA profile and compare this with miRNAs measured in human BAT. To achieve this we firstly established a mouse BAT enriched-miRNA profile by comparing miRNAs expressed in mouse BAT, white adipose tissue and skeletal muscle. Following this the BAT-enriched miRNAs predicted to target genes potentially involved in growth and development were identified.

**Methods:**

MiRNA levels were measured using PCR-based miRNA arrays. Results were analysed using ExpressionSuite software with the global mean expression value of all expressed miRNAs in a givensample used as the normalisation factor. Bio-informatic analyses was used to predict gene targets followed by Ingenuity Pathway Analysis.

**Results:**

We identified 35 mouse BAT-enriched miRNAs that were predicted to target genes potentially involved in growth and development. We also identified 145 miRNAs expressed in both mouse and human BAT, of which 25 were enriched in mouse BAT. Of these 25 miRNAs, miR-20a was predicted to target MYF5 and PPARγ, two important genes involved in brown adipogenesis, as well as BMP2 and BMPR2, genes involved in white adipogenesis. For the first time, 69 miRNAs were identified in human BAT but absent in mouse BAT, and 181 miRNAs were expressed in mouse but not in human BAT.

**Conclusion:**

The present study has identified a small sub-set of miRNAs common to both mouse and human BAT. From this sub-set bioinformatics analysis suggested a potential role of miR-20a in the control of cell fate and this warrants further investigation. The large number of miRNAs found only in mouse BAT or only in human BAT highlights the differing molecular profile between species that is likely to influence the functional role of BAT across species. Nevertheless the BAT-enriched miRNA profiles established in the present study suggest targets to investigate in the control BAT development and EE.

**Electronic supplementary material:**

The online version of this article (doi:10.1186/s12864-015-2045-8) contains supplementary material, which is available to authorized users.

## Background

Obesity is a world-wide epidemic that increases the risk of cardiovascular disorders, cancer and type 2 diabetes (T2D) [1]. Obesity results from an energy imbalance whereby energy intake (EI) exceeds energy expenditure (EE). Strategies to decrease obesity have been orientated towards a reduction in EI, unfortunately without success [2]. Consequently, new therapeutic strategies aimed at targeting EE are being investigated. Discovery of active human brown adipose tissue (BAT) has renewed interest in the possibility that it may regulate metabolism and EE in humans [3–5].

Skeletal muscle is another tissue that plays an important role in regulating EE and several lines of evidence suggest that skeletal muscle and BAT share a common lineage [6, 7]. microRNAs (miRNAs) are short non-coding RNAs that regulate gene expression and tissue development [8, 9]. Muscle-enriched microRNAs including miR-1, −133a and −206, are also expressed in murine brown pre and mature adipocytes, but not in white adipocytes [10]. The PRD1-BF-1-RIZ1 homologous domain protein containing protein-16 (PRDM16), a BAT-enriched protein controls the differentiation between BAT and skeletal muscle by forming a transcriptional complex with CCAAT/Enhancer binding protein β (C/EBPβ) [11–13]. Understanding the mechanisms controling BAT differentiation may identify potential therapeutic targets to enhance BAT-controlled metabolism and to increase EE.

MiRNA screenings and bioinformatics analyses have identified potential gene targets and biological functions controlled by certain miRNAs in tissues and diseases. Sun et al. observed 91 differentially expressed miRNAs when comparing mouse BAT, white adipose tissue (WAT) and skeletal muscle [14]. The oligonucleotide microchip technology used in this study allowed for the screening of only ~350 miRNAs however is not as sensitive as quantitative real time-polymerase chain reaction (RT-PCR) array technology [15]. Investigation of a broader number of BAT miRNA targets using highly sensitive qPCR-array methods will provide a more comprehensive overview of the potential miRNAs regulating BAT development and function. Several miRNAs controlling mouse brown adipocyte development and function have been identified in mice, including miR-27, −34a, −133, −155, −182, −193b-365, −196, −203 and miR-378 [14, 16–23]. MiR-27 is decreased in mouse BAT following cold exposure and during brown adipogenesis [22]. As a consequence, several of the miR-27 target genes, including Prdm16, peroxisome proliferator-activated receptor alpha (Pparα), cAMP response element-binding protein (Creb) and peroxisome proliferator-activated receptor gamma coactivator 1-beta (Pgc1β) are upregulated, and enhance brown adipogenesis. MiR-34a inhibits beige and brown fat formation by targeting fibroblast growth factor-21 (Fgf21) and sirtuin 1 (Sirt1), two known activators of WAT browning [20]. miR-133 directly targets the 3’-UTR of *Prdm16* [19] and controls the differentiation of satellite cells within skeletal muscle towards an adipogenic or myogenic phenotype [17, 19]. While the cluster miR-193–365 is up-regulated by Prdm16, partially through Pparγ [14], they are not required for brown fat development and function [24]. MiR-182 and miR-203 are BAT-specific miRNAs, essential for the maintenance and differentiation of brown adipocytes *in vivo* [21]. Finally, miR-378 increases brown fat mass and as a consequence, suppresses development of beige adipocytes in subcutaneous WAT [23]. However it is unknown if these miRNAs are expressed in human BAT.

BAT plays a critical role in the regulation of energy balance and temperature in rodents and newborns [25–27], however its role in human adult metabolism remains equivocal. While mice still remain the investigative of model of choice to understand the role and regulation BAT, studies establishing the similarities and differences in the molecular profile of mouse and human BAT are required.

Therefore the primary aims of this study were to (1) identify BAT-enriched miRNAs by comparing miRNA expression mouse BAT, skeletal muscle and WAT using PCR-based miRNA arrays (2); predict the BAT-enriched miRNA target genes potentially involved in growth, proliferation and development; (3) compare the miRNA profiles of mouse and human BAT.

## Results

### Mouse BAT, WAT and skeletal muscle tissues

#### miRNA array analysis on mouse tissues

**Fig. 1:**
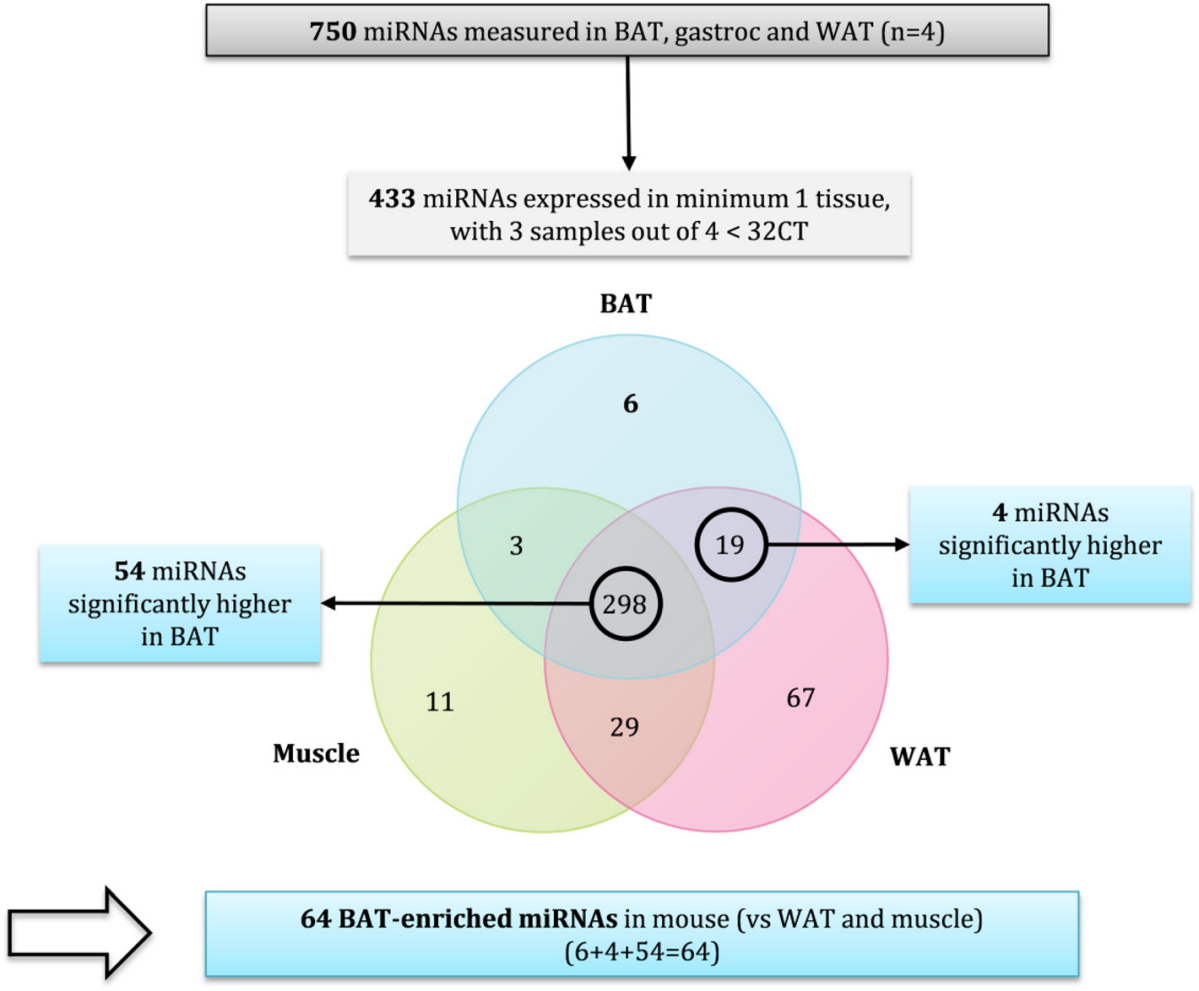
Flow chart of the miRNA array analysis from mouse tissues

**Fig. 2:**
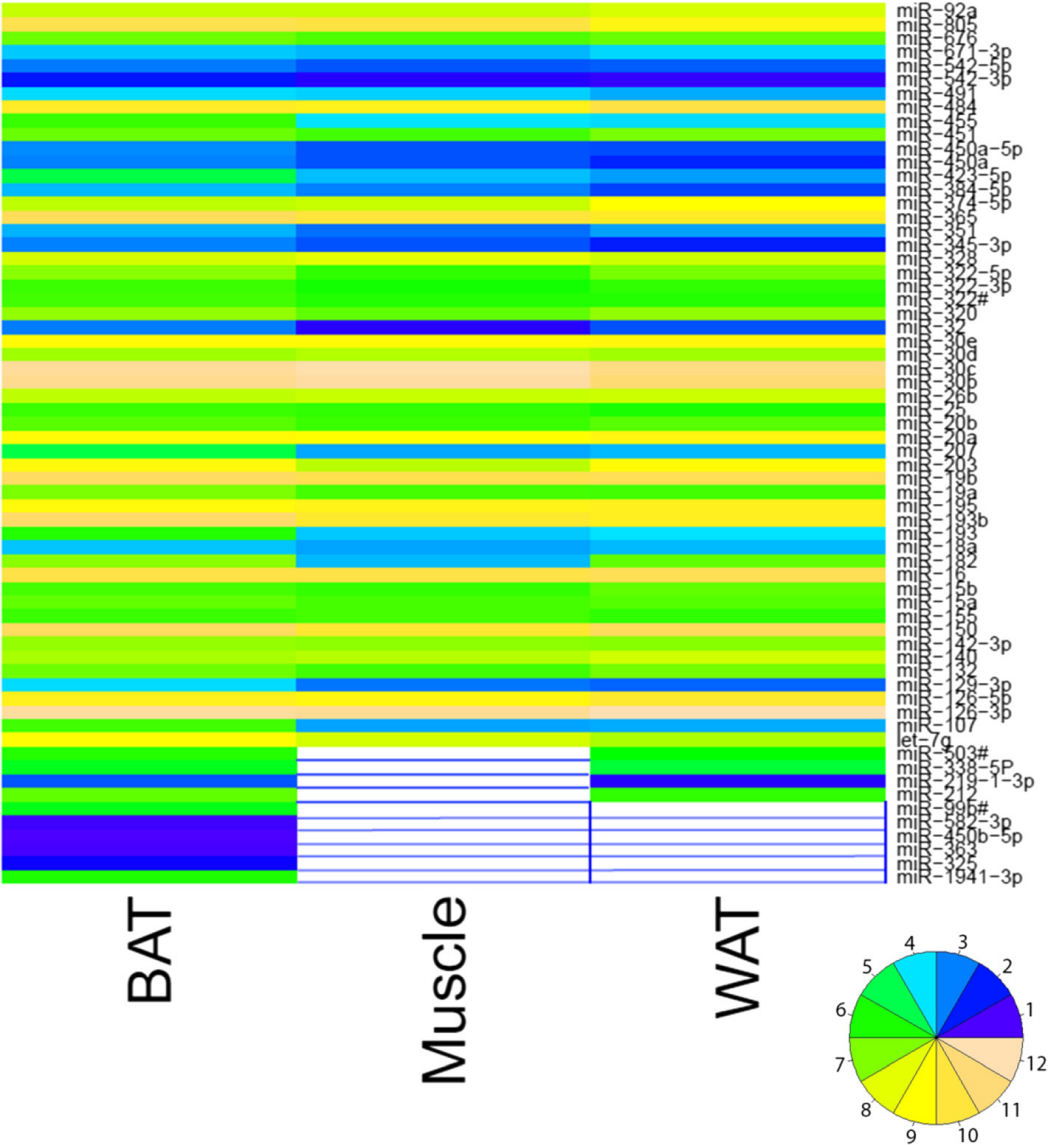
Heat map of the 64 miRNAs specifically and highly expressed in mouse BAT as compared with muscle and WAT. Values 1 and 12 represent the lowest and highest values, respectively. White values represent non-expressed miRNAs

To define miRNAs enriched in mouse BAT, comparisons were made between BAT, skeletal muscle (gastrocnemius) and WAT as described in the flowchart (Fig. 1). From the 750 miRNAs profiled 433 miRNAs were expressed at least in one tissue. The Venn diagram represents the tissue distribution of these 433 miRNAs. Six miRNAs were exclusively expressed in BAT. Nineteen miRNAs (circled) were expressed only in BAT and WAT, with four of these significantly higher in BAT tissue (*p* < 0.05). Three miRNAs were expressed only in BAT and muscle and at similar levels. There were 298 miRNAs (circled) commonly expressed in the three tissues, of which 54 were significantly higher in BAT compared to WAT and muscle (*p* < 0.05). When adding all the exclusively and highly expressed miRNAs found in BAT, 64 BAT-enriched miRNAs were obtained as shown in the heat map below (Fig. 2).

Eleven miRNAs were exclusively expressed in muscle, 67 miRNAs were exclusively expressed in WAT, while another 29 of miRNAs were expressed in both muscle and WAT only (Additional file 1). A more detailed explanation of this analysis can be found in Additional file 2.

#### Bio-informatic analyses on mouse BAT-enriched miRNAs

To identify miRNAs potentially involved in BAT development a microRNA target filter was performed using IPA software (Ingenuity®Systems, www.ingenuity.com). Of the 64 BAT-enriched miRNAs, 35 miRNAs were predicted to target 859 genes potentially involved in cellular growth, proliferation and development (Additional file 3). Table 1 presents these 35 miRNAs as well as the number of predicted genes targeted by each miRNA. miR-15a was predicted to target the most genes involved in cellular growth, proliferation and development pathways.

**Table 1:** Highly expressed miRNAs in mouse BAT. Number of targets predicted to regulate growth, proliferation and development. Relative expression values are arbitrary units (A.U.) and have been calculated relative to mouse miR-15a

miRNA	Number of targeted mRNAs	Relative expression (A.U)	miRNA	Number of targeted mRNAs	Relative expression (A.U.)
miR-15a	184	1.0	miR-140	48	2.7
miR-20a	149	8.1	miR-150	45	69.4
miR-19a	141	1.5	miR-193	39	0.4
miR-30b	139	239.2	miR-328	39	5.06
miR-182	135	1.8	miR-542-3p	39	0.004
let-7g	133	7.9	miR-491	38	0.07
miR-203	112	9.1	miR-18a	37	0.05
miR-351	108	0.0	miR-455	33	0.54
miR-26b	106	3.8	miR-484	11	17.6
miR-25	100	0.6	miR-451	10	1.10
miR-320	97	2.0	miR-129-3p	9	0.1
miR-107	92	0.6	miR-126-3p	6	337.2
miR-374-5p	89	3.8	miR-450a-5p	3	0.0
miR-155	82	0.6	miR-671-3p	3	0.1
miR-132	75	1.2	miR-345-3p	2	0.1
miR-423-5p	58	0.1	miR-582-3p	2	0.0
miR-142-3p	55	2.1	miR-338-5P	1	0.2
miR-365	49	67.1			

### Comparison of miRNA expressed in human and mouse BAT

**Fig. 3:**
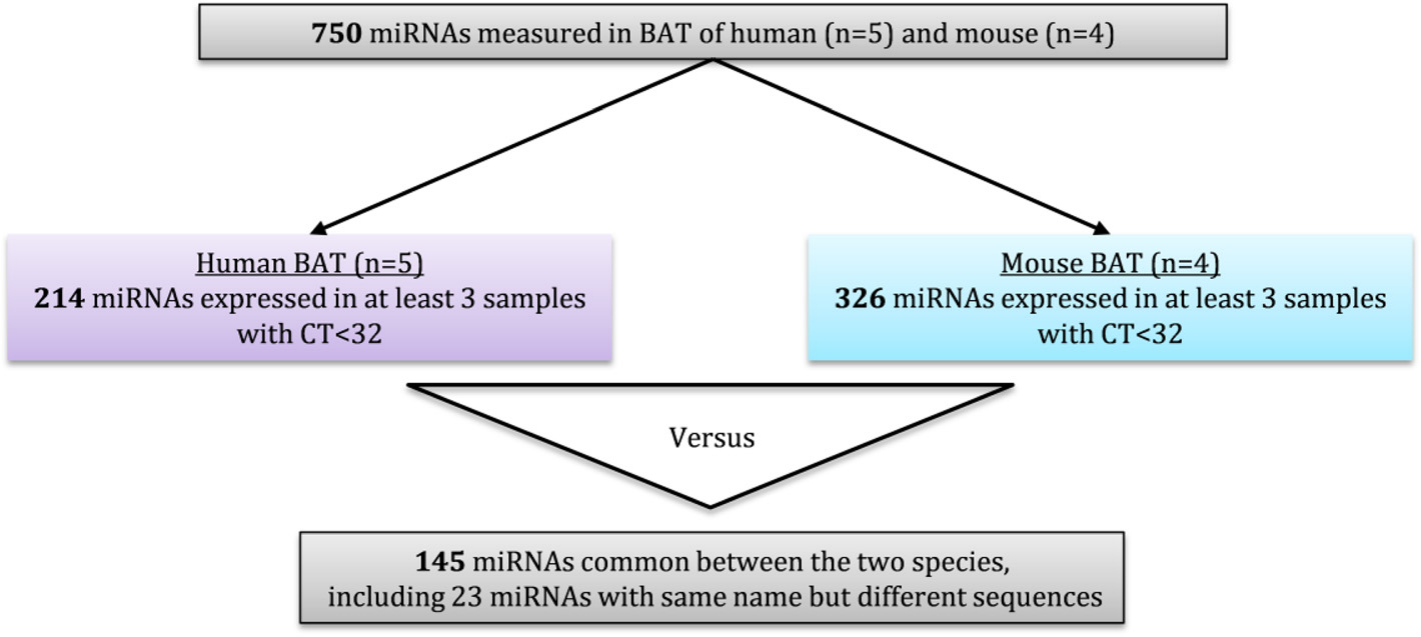
Flow chart of the miRNA array analysis for the comparisons of human and mouse BAT

To establish if human and mouse BAT express similar BAT-enriched miRNAs, miRNA array analysis was performed on 5 human and 4 mouse BAT samples. Data were analysed as described in the flowchart (Fig. 3). Of the 750 miRNAs measured 214 miRNAs and 326 miRNAs were expressed in a minimum of 3 human BAT samples and 3 mouse BAT samples, respectively. Of these miRNAs, 145 were expressed in both human and mouse BAT (Additional file 4), including 23 miRNAs that had the same name but their sequences differed by few nucleotides between the species (i.e. miR-155, miR-193b, miR-455). Therefore, 69 miRNAs were expressed in human BAT but not mouse BAT, while 181 miRNAs were expressed in mouse BAT but not human BAT.

**Fig. 4:**
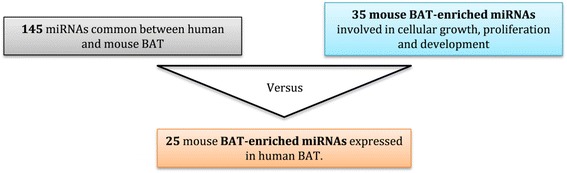
Flow chart of the miRNA analysis for the comparisons between human and mouse BAT-enriched miRNAs

**Fig. 5:**
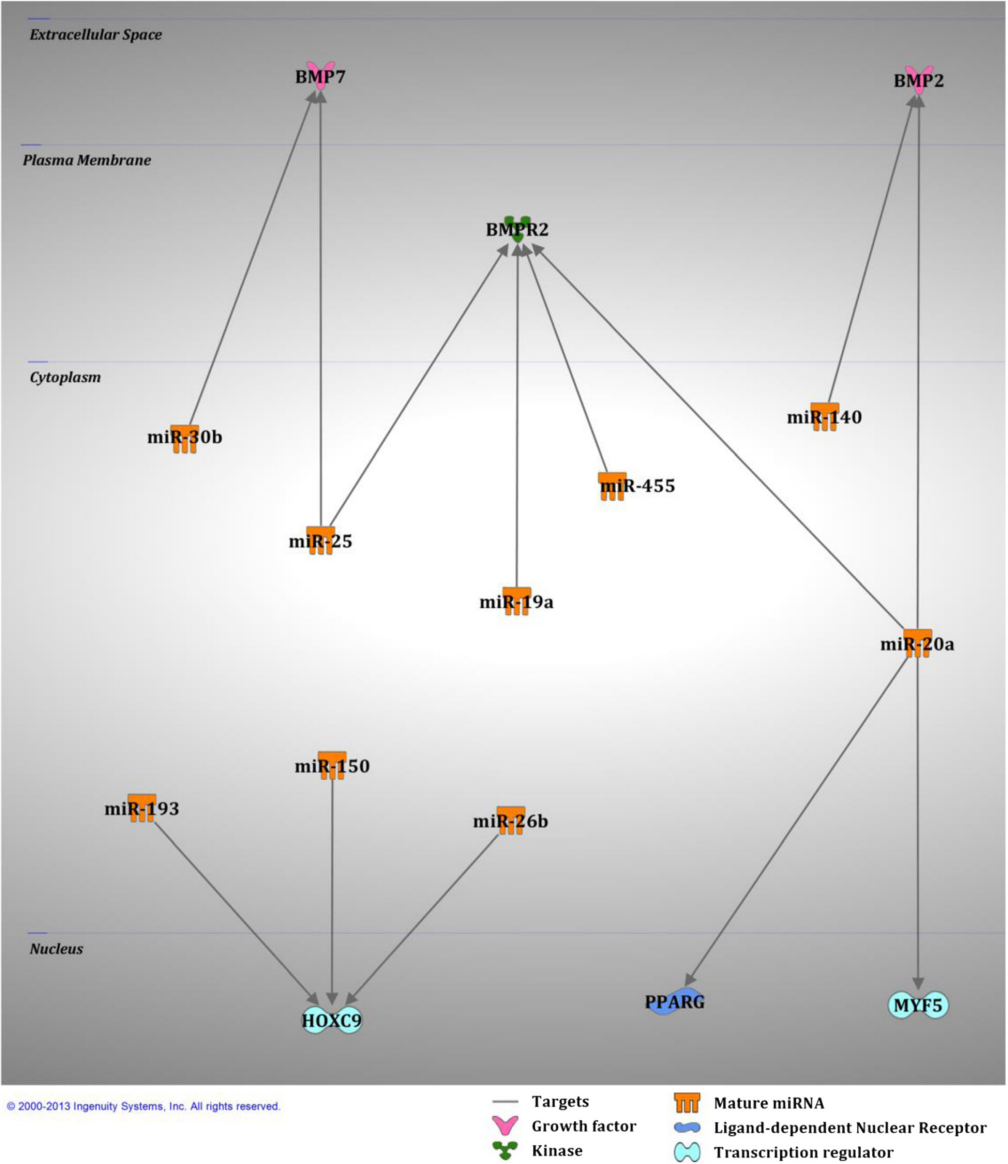
Selected miRNAs common to human and mouse BAT and their gene targets known to be involved in brown adipogenesis

As shown in the flowchart in Fig. 4 a comparison was made between the 145 miRNAs common to both human and mouse BAT and with the 35 mouse BAT-enriched miRNAs identified from the bio-informatic analyses in Table 2. Twenty five of the 35 mouse BAT-enriched miRNAs were also expressed in human BAT and were predicted to regulate 788 genes involved growth, proliferation and development pathways (Additional file 5). Ten miRNAs enriched in mouse BAT were not expressed in human BAT. Of the 25 BAT-enriched miRNAs commonly expressed in human and mouse BAT, 10 had previously been predicted to target gene involved in brown adipogenesis. These 10 miRNAs and their predicted regulatory network are presented in Fig. 5. *Bone morphogenetic protein 2* (*BMP2*) and *BMP7* are predicted to be targeted by miR-20a,-140 and miR-25, −30b, respectively. *Bone morphogenetic protein receptor 2* (*BMPR2*) is known to be targeted by miR-19a, −20a and miR-25 [28] and predicted to be targeted by miR-455. *Homeobox C9* (*HOXC9*) is predicted to be targeted by miR-193, 150 and -26b. Finally, *PPARγ* is known to be targeted by miR-20a [29] and *myogenic factor 5 (MYF5)* is predicted to be targeted by miR-20a.

**Table 2:** miRNA expression in both human and mouse BAT. Number of targets predicted to regulate growth, proliferation and development. Relative expression values are arbitrary units (A.U.) and have been calculated relative to human miR-15a

miRNA	Number of targeted mRNAs	Relative expression (A.U)	miRNA	Number of targeted mRNAs	Relative expression (A.U)
miR-15a	184	1.0	miR-365	49	9.2
miR-20a	149	13.2	miR-140	48	7.2
miR-19a	141	12.7	miR-150	45	101.4
miR-30b	139	26.2	miR-193	39	6.8
let-7g	133	15.1	miR-328	39	0.4
miR-203	112	0.3	miR-491	38	0.5
miR-26b	106	15.5	miR-18a	37	0.2
miR-25	100	0.3	miR-455	33	0.4
miR-320	97	44.3	miR-484	11	65.6
miR-155	82	4.6	miR-451	10	1.9
miR-132	75	6.8	miR-126-3p	6	1438.0
miR-423-5p	58	0.4	miR-338-5P	1	1160.2
miR-142-3p	55	2.7

## Discussion

The recent discovery of active BAT in human adults [30–35] has opened a new field of investigation for the treatment of obesity [3–5]. Therefore, understanding the molecular mechanisms, regulating human BAT development may identify novel therapeutic strategies to increase energy expenditure. miRNAs are important molecular switches that display high levels of tissue enrichment and can control cell differentiation and tissue growth and development. Studies have investigated miRNAs in mouse BAT [10, 14, 16–24], however their expression levels have not been validated in human BAT. The present study measured and compared the expression of miRNAs highly enriched in mouse and human BAT, as well as predicting their gene targets potentially involved in BAT growth and development. Several novel observations were made. Firstly, 64 miRNAs were exclusively or highly expressed in mouse BAT in comparison to mouse skeletal muscle and WAT. Secondly, of these 64 BAT-enriched miRNAs, 35 were predicted to regulate genes involved in cellular growth, proliferation and development. Thirdly, 145 miRNAs were found to be commonly expressed in human and mouse BAT. Finally, 25 of these 145 miRNAs were also identified in the list of 35 mouse BAT-enriched miRNAs potentially involved in cellular growth, proliferation and development.

Identifying the molecular mechanisms (i.e. miRNAs) controlling BAT development has become a priority to develop therapeutic strategies in the fight against obesity. While BAT shares a common origin with skeletal muscle [6, 7], studies in mouse WAT recently discovered a phenomenon called “browning” that involves the formation of brown adipocytes in WAT after cold exposure or β-adrenergic stimulation [36]. Therefore, it was of interest to compare these three mouse tissues in order to identify BAT-enriched miRNAs. In the present study, qPCR-based miRNA screening of ~750 miRNAs identified 64 miRNAs exclusively or highly expressed in mouse BAT, in comparison to skeletal muscle and WAT. Consistent with a similar study [14], we identified that ~22% of the miRNAs commonly expressed between the three tissues were significantly higher in BAT. Of the 64 miRNAs observed to be enriched in mouse BAT in the present study, only 15 have previously in BAT [14].

miRNAs regulate various component of cellular networks and maintain cellular homeostasis [37]. Therefore it was of interest to select BAT-enriched miRNAs that were predicted to target genes specifically involved in cellular growth, proliferation and development and as such may also control BAT development. Thirty-five of the 64 miRNAs were predicted to target 859 genes involved in growth and development pathways. In cultured adipocytes, global deep sequencing identified 16 and 12 miRNAs that were differentially regulated in mouse primary pre-adipocytes when compared with differentiated mature brown adipocytes in vitro*,* [18]. Of the miRNAs upregulated in differentiated mature brown adipocytes, miR-142-3p and -19a were also up-regulated in the present list of 35 BAT-enriched miRNAs predicted to target genes involved in growth and differentiation. This supports a potential role of these miRNAs during BAT development in mice. While miR-155 and −328 were down-regulated in mature brown adipocytes when compared to pre-adipocytes, they were identified as being upregulated in our list of 35 BAT-enriched miRNAs. These discrepancies may be indicative of the differences between mature brown adipocytes *in vitro* and mature BAT.

Most studies have used mice as a model to study BAT development and function, due to the previously held view that adult humans did not possess BAT, as well as the limited access to human BAT from new born babies. As such, no study has looked at the miRNA profile of human BAT. It was therefore of interest to compare miRNA profiles of mouse and human BAT to establish the potential molecular similarities and differences between the two species. These comparisons also provide a snapshot of the potential to translate molecular observations from mice to humans. For the first time, a miRNA screening revealed the presence of 214 miRNAs expressed in human BAT, including 145 commonly expressed in mouse BAT. This screening also showed that 69 miRNAs expressed in human BAT were not expressed in mouse BAT and therefore were not taken into consideration when comparisons were made with the mouse model. Further investigations of these 69 miRNAs will be required to identify their potential unique role in human BAT development and function. In contrast, 181 miRNAs in mouse BAT were not present in human BAT, suggesting that translating results from mouse to human BAT needs careful consideration.

Of the 145 miRNAs common to both mouse and human BAT, 25 were identified in the list of 35 mouse BAT-enriched miRNAs potentially targeting genes involved in cellular growth, proliferation and development. 788 genes involved in these pathways were predicted to be targeted by these 25 miRNAs. In this present study, miR-20a was the only miRNA predicted to target *MYF5* and *PPARγ* (a previously experimentally observed target [38]), two important factors directing cell fate towards a brown fat phenotype [11]. In contrast, miR-20a is also predicted to target *BMP2* and *BMPR2*, a growth factor and receptor increased in white fat differentiation [39]. These findings suggest that miR-20a may have the capacity to control cell fate toward a brown or white fat phenotype. Furthermore, miR-20a was the miRNA targeting the second most numbers of genes (*n* = 149) involved in growth and development, highlighting its potential importance in these pathways. To date, miR-20a has not been studied in relation to BAT growth and development and therefore requires further investigation.

miR-155, highly expressed in brown pre- adipocytes when compared to differentiated mature brown adipocytes, inhibits brown adipogenesis by targeting *C/EBPβ* [18], a transcription factor forming a complex with PRDM16 during brown adipogenesis [13]. In contrast to these observations, miR-155 was identified as a highly enriched miRNA in human and mouse BAT. This observation suggests that human BAT may be comprised of a mixed composition of cells with different stages of cell maturation. However, the presence of miR-20a, −25 and -30b, all predicted to inhibit expression of genes involved in brown fat development, may prevent the development of a “classical” brown fat phenotype in human BAT. Furthermore, miR-182 and −203 were also part of the top ten miRNAs targeting the most genes involved in growth and development in mouse BAT, suggesting their potentials roles in brown adipocyte development [21]. However, only miR-203 was conserved in human BAT. The absence of miR-182 expression in human BAT might contribute to differences between mouse and human BAT development and function. A gene signature developed in human BAT identified a profile closely resembling a brite/beige phenotype [40, 41]. This phenotype is supported by the absence of miR-378, a key regulator controlling classical BAT-specific expansion and obesity resistance [23]. However the phenotypical signature may depend on the depth of the tissue taken with superficial tissue representing a more WAT-like signature while deeper tissue representing a more classical BAT-like signature [42].

The miR-193b-365 cluster and miR-455 are known to regulate mouse brown fat development and were identified in our selection of 25 BAT-enriched miRNAs common to mice and humans. Inhibition of miR-193a/b and/or miR-365 in mouse primary brown pre-adipocytes impairs brown adipogenesis *in vitro* [14]. However, miR-193b null mice, that are also deficient in miR-365, have normal BAT development, differentiation and function [24]. miR-193b null mice have elevated levels of miR-455 suggesting a compensatory effect in the absence of the miR-193b-365 cluster. miR-455 expression levels are also increased in mature murine brown adipocytes and positively correlate with uncoupling protein 1 (UCP1) levels suggesting a potential metabolic role in brown adipocytes [10]. miR-19a, −140, −150 and -26b are predicted to target white fat-related genes (*BMP2* or *BMPR2* or *HOXC9*) [39, 41] and therefore may have a role in selecting cell fate toward brown adipogenesis.

In the present study BAT from newborns was used to establish the human BAT miRNA profile. How precisely these findings translate to the human adult is presently unknown. Human newborns and adults have a similar distribution of BAT depots [43]. In newborns and infants BAT is mostly located in the interscapular, cervical and perirenal regions [43, 44]. In human adults the major depots of metabolically active BAT have been identified in the cervical, supraclavicular, axillary and paravertebral regions [30, 32, 33]. The location and quantity of human BAT alters with age, which may indicate an age-dependent change in its primary role; presently the precise functional role of adult BAT is yet to be determined. However, when the hands of both infants and adults are cooled there is a 0.3-0.7°C increase in subcutaneous cervical BAT, albeit to a lesser degree in older subjects [45]. This suggests that both infant and adult BAT may respond in a similar way to some external stress signals. Several studies have tried to develop a mRNA profile to establish how similarly infant and adult human BAT [40, 42] and brown adipocytes [44, 46] mimic the “classical” rodent BAT on a molecular level. Depending on the depth and location of the tissue analysed, infant and adult human BAT possess gene signatures with a mixture of “classical” BAT and “beige-like” adipocytes. The present study analysed newborn BAT taken from regions where active BAT is also found in the human adult. Therefore, it is most likely that the newborn human BAT miRNA profile established here would represent an adult profile with a mixture of “classic” BAT and/or “beige-like” adipocytes. Investigations are still required to clearly identify the mRNA and the miRNA species that are truly specific to brown and beige tissue; a task that that may complicated by the different levels of heterogeneity within BAT tissue depots.

## Conclusion

This study has identified 64 miRNAs enriched in mouse BAT when compared to skeletal muscle and WAT, including 35 miRNAs specifically involved in cellular growth, proliferation and development. Furthermore, for the first time a comparison between human and mouse BAT revealed the presence of 145 commonly expressed miRNAs in BAT from both species. These included 25 miRNAs specifically expressed in mouse BAT and involved in cellular growth, proliferation and development. The identification of BAT-enriched miRNAs, conserved in both mouse and human BAT, such as miR-20a, may be a common factor controlling BAT development. Several of the newly identified miRNAs, common to mouse and human BAT, should be evaluated for their direct role in brown adipogenesis.

## Methods

### Tissue collection

The interscapular BAT, gastrocnemius and visceral WAT from 8 week old male C57BL/6J mice were donated by Professor Matthew Watt, Monash University. The tissue provided was approved by the School of Biomedical Sciences Animal Ethics Committee (SOBSA/2009/22) Monash University, Australia. RNA samples from human BAT, obtained from newborn humans aged between 10.6 ± 4.3 days, were donated by Professor Vicente Gilsanz from the Children^’^s Hospital Los Angeles (CHLA), USA. The RNA samples were originally obtained as a component of a previously published study [40]. This previously published human study was approved by the Institutional Review Board of the CHLA (CCI-10-00073). The Deakin University Human Research Ethics Committee Executive also reviewed the project (CCI-10-00073) and acknowledged the approval granted by the Institutional Review Board of the CHLA and allocated the project reference number 2015–189. Written informed consent was obtained by all parent/legal guardian(s) in the presence of a witness authorizing post-mortem examination and removal of tissues. Authorization was also given for the samples to be retained and preserved and to be used for diagnostic, therapeutic and other scientific purposes as deemed appropriate by the examining physician or surgeon. The research was carried out on in compliance with the Helsinki Declaration. The samples were surgically removed from the subcutaneous supraclavicular/supracervical area, intra-abdominal area or retroperitoneal area during post-mortem examination 24h after death and have been used previously of mRNA measurements [40]. miRNAs are exceptionally stable post-mortem [47] and therefore the miRNA profile generated would be representative of that observed in a living human. All tissue samples were snapped frozen in liquid nitrogen and kept at −80°C until required for RNA extraction.

### RNA extractions

#### Mouse tissues

Approximately 20 mg of BAT and gastrocnemius and 100 mg of WAT were homogenised in 500 μl of Tri-Reagent® Solution (Life Technologies, CA, USA), using the MagNA Lyser (Roche, Basel, Switzerland) for 20s at a speed setting of 5500 revolutions per minute (rpm). After transferring the homogenate into a fresh tube, RNA extraction was completed according to the manufacturer’s protocol. Quality of the RNA was assessed using a Nanodrop 1000 Spectrophotometer (Thermo Fisher Scientific, MA, USA) and only samples with an O.D. 260/280 ratio >1.9 were used for the analyses.

#### Human tissues

Approximately 20 mg of BAT were homogenised in 300 μl of the denaturation solution from the TōTALLY RNA kit™ (Life Technologies, CA, USA), using the MagNA Lyser (Roche, Basel, Switzerland) for 20s at a speed setting of 5500rpm. After transferring the homogenate into a fresh tube, RNA extraction was completed according to the manufacturer’s protocol. Quality of the RNA was assessed using a Nanodrop 1000 Spectrophotometer (Thermo Fisher Scientific, MA, USA) and only samples with an O.D. 260/280 ratio >1.9 were used for the analyses.

### Megaplex reverse transcription and miRNA arrays

#### Mouse and human tissues

Rodent Megaplex™ Primer Pools and TaqMan® Array miRNA Cards A version 2.0 and B version 3.0 (Life Technologies, CA, USA) were used to reverse transcribe and amplify ~750 miRNAs according to the manufacturer’s protocol. This was performed on 4 BAT, 4 gastrocnemius and 4 WAT samples. Human Megaplex™ Primer Pools and TaqMan® Array miRNA Cards A version 2.1 and B version 3.0 (Life Technologies, CA, USA) were used to reverse transcribe and amplify ~750 miRNAs according to the manufacturer’s protocol. This was performed on human tissues from 5 different individuals. All cards were run on the 7900HT Fast Real-Time PCR System machine at Monash Institute of Medical Research (MIMR, Clayton, VIC, Australia). Results were analysed using ExpressionSuite software v1.0.1 (Life Technologies, CA, USA). The global mean expression value of all expressed miRNAs in a given samples was used as the normalisation factor in this study [48].

### Bio-informatics

Bio-informatic analyses were performed on specific lists of miRNAs to determine their predicted gene targets. Data were analysed through the use of IPA (Ingenuity®Systems, www.ingenuity.com) and more specifically, the microRNA Target filter tool. Filters were used to refine the analysis based on experimentally observed and high predicted gene targets only in the column “confidence”, as well as gene involved in cellular growth, proliferation and development pathways only.

#### Statistics

For the mouse samples, miRNAs were considered expressed in a given tissue when a minimum of 3 out of the 4 samples analysed had CT values below 32, as suggested in the manufacturer’s protocol. miRNAs expressed in 2 tissues only were compared using an unpaired t-test following log transformation of their expression values. For the miRNAs expressed in the 3 tissues (BAT, gastrocnemius and WAT), statistical analysis was performed using STATA data analysis and statistical software using a Kruskal–Wallis test. When a significant difference was detected a two-sample Wilcoxon-rank sum test was used to locate the differences. A p-value of <0.05 was considered as significant for all analyses.

For the human samples, miRNAs were considered expressed in a given tissue when a minimum of 3 samples (out of 5) had CT values below 32, as mentioned in the manufacturer’s protocol.

### Availability of supporting data

The data sets supporting the results of this article are included within the articles (and its additional files).

## Additional files

Additional file 1:
**Muscle-enriched and WAT-enriched miRNAs, and miRNAs commonly enriched in mouse muscle and WAT.** (PDF 254 kb) Additional file 2:
**Flow chart of the miRNA array analysis in mouse tissues.** (PDF 539 kb) Additional file 3:
**Table of the 35 mouse BAT-enriched miRNAs and their predicted gene targets involved in cellular growth, proliferation and differentiation.** (PDF 447 kb) Additional file 4:
**Table of the 145 miRNAs commonly expressed in both human and mouse BAT.** (PDF 310 kb) Additional file 5:
**Table of the 25 mouse BAT-enriched miRNAs in human BAT and their predicted gene targets involved in cellular growth, proliferation and differentiation.** (PDF 429 kb)

## Abbreviations

AU: Arbitrary unit
BAT: Brown adipose tissue
BMP2: Bone morphogenetic protein 2
BMPR2: Bone morphogenetic protein receptor 2
C/EBPβ: CCAAT/Enhancer binding protein β
Creb: cAMP response element-binding protein
EI: Energy intake
EE: Energy expenditure
Fgf21: Fibroblast growth factor-21
HOXC9: Homeobox C9
miRNA / miR: microRNA
MYF5: Myogenic factor 5
Pgc1β: Peroxisome proliferator-activated receptor gamma coactivator 1-beta
Pparα: Peroxisome proliferator-activated receptor alpha
PRDM16: PRD1-BF-1-RIZ1 homologous domain protein containing protein-16
RNA: Ribonucleic acid
RT-PCR: Real time-polymerase chain reaction
Sirt1: Sirtuin 1
T2D: Type 2 diabetes
WAT: White adipose tissue
