# Supplementary material for: Comparative analysis of microRNA expression in mouse and human brown adipose tissue
Source: BMC Genomics. 2015 Oct 19;16:820. doi: 10.1186/s12864-015-2045-8 (PMC4617708; doi:10.1186/s12864-015-2045-8)
Supplement: Additional file 1: — Muscle-enriched and WAT-enriched miRNAs, and miRNAs commonly enriched in mouse muscle and WAT. (PDF 254 kb) [file 12864_2015_2045_MOESM1_ESM.pdf]

Additional file 1: Muscle-enriched and WAT-enriched miRNAs, and miRNAs commonly enriched in mouse muscle and WAT.

Mouse tissues			
**Muscle-enriched miRNA ID**	**WAT-enriched miRNA ID (1/2)**	**WAT-enriched miRNA ID (2/2)**	**Muscle & WAT-enriched miRNA ID**
miR-1193	let-7a#	miR-297a#	miR-136#
miR-1943	let-7d#	miR-299	miR-143
miR-206	let-7g#	miR-29a#	miR-196a
miR-211	miR-10b#	miR-30b#	miR-213
miR-466b	miR-1186	miR-30c-2#	miR-214#
miR-489	miR-1188	miR-31#	miR-299-5p
miR-493	miR-1191	miR-326	miR-340
miR-493-3p	miR-1195	miR-327	miR-421
miR-673-3p	miR-125b#	miR-33a#	miR-10b
miR-675-3p	miR-1306	miR-344	miR-1198
miR-704	miR-144	miR-345	miR-1839-5p
	miR-147	miR-347	miR-186#
	miR-148a#	miR-34b-5p	miR-1897-5p
	miR-15a#	miR-362-5p	miR-1905
	miR-15b#	miR-376a#	miR-1954
	miR-17#	miR-376a#	miR-196b
	miR-1894-3p	miR-377	miR-2182
	miR-1896	miR-465a-3p	miR-2183
	miR-1940	miR-465b-5p	miR-337
	miR-1957	miR-466a-3p	miR-369-3p
	miR-1961	miR-466g	miR-376b#
	miR-1981	miR-466J	miR-504
	miR-200a#	miR-467a	miR-539
	miR-200b	miR-467b	miR-590-5p
	miR-200c	miR-485-3p	miR-615-5p
	miR-20a#	miR-632	miR-665
	miR-216b	miR-669n	miR-696
	miR-217	miR-676#	miR-29c#
	miR-27a#	miR-712	miR-350
	miR-28-3p	miR-743a	
	miR-292-3p	miR-744#	
	miR-293	miR-878-3p	
	miR-295	miR-99a#	
	miR-296-3p		
