# Supplementary material for: Comparative analysis of microRNA expression in mouse and human brown adipose tissue
Source: BMC Genomics. 2015 Oct 19;16:820. doi: 10.1186/s12864-015-2045-8 (PMC4617708; doi:10.1186/s12864-015-2045-8)
Supplement: Additional file 2: — Flow chart of the miRNA array analysis in mouse tissues. (PDF 539 kb) [file 12864_2015_2045_MOESM2_ESM.pdf]

Additional file 2: Flow chart of the miRNA array analysis in mouse tissues.

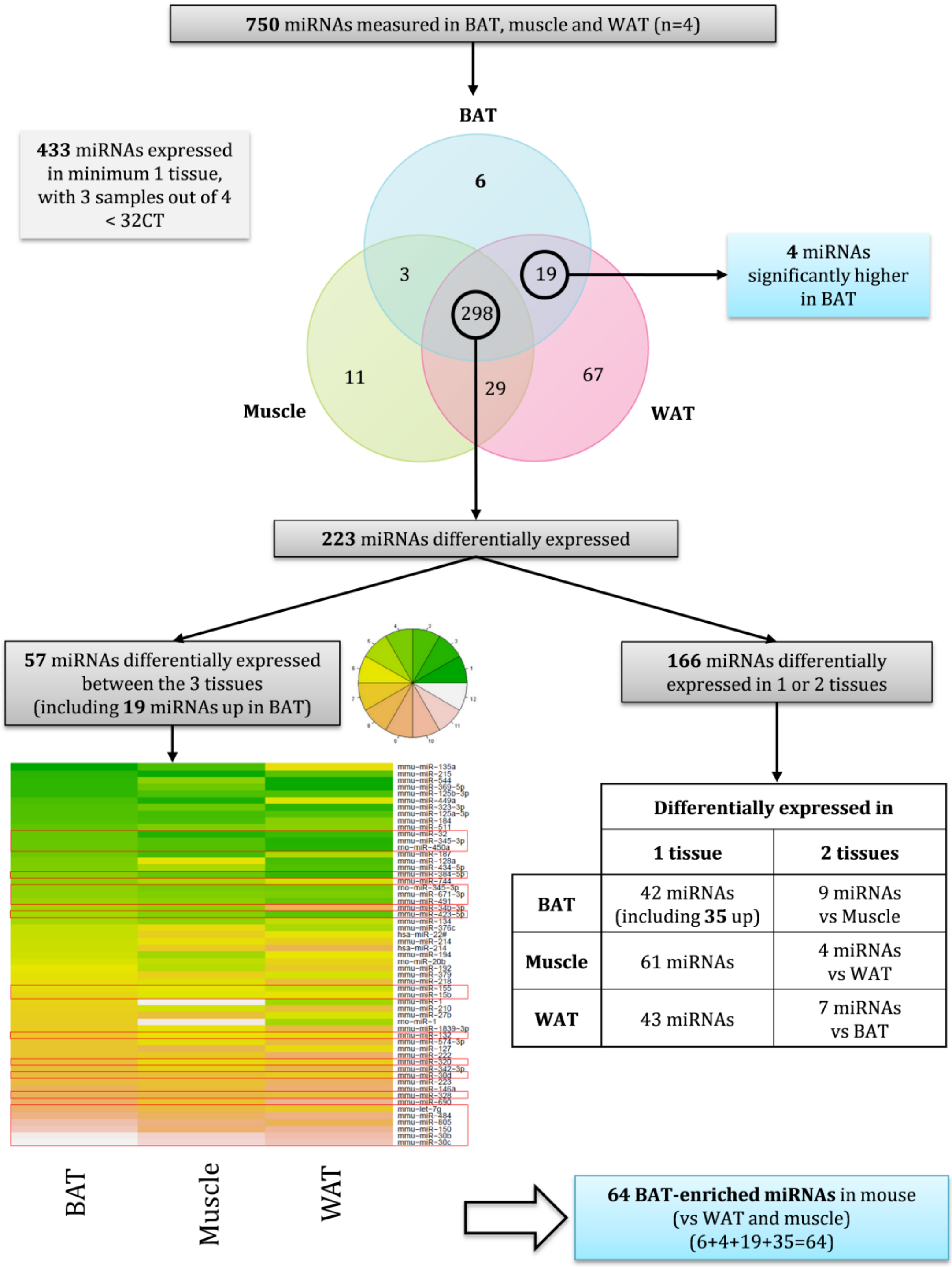

	Differentially expressed in	
	**1 tissue**	**2 tissues**
**BAT**	42 miRNAs (including **35** up)	9 miRNAs vs Muscle
**Muscle**	61 miRNAs	4 miRNAs vs WAT
**WAT**	43 miRNAs	7 miRNAs vs BAT

To define miRNAs enriched in mouse BAT, comparisons were made between BAT, muscle and WAT as described in the flowchart. From the 750 miRNAs measured 433 miRNAs were expressed at least in one tissue. The Venn diagram represents the tissue distribution of these 433 miRNAs. Six miRNAs were exclusively expressed in BAT. Nineteen miRNAs (circled) were expressed only in BAT and WAT, with 4 of these significantly higher in BAT

tissue. Three miRNAs were expressed only in BAT and muscle, but none were significantly elevated in BAT. There were 298 miRNAs (circled) commonly expressed in the 3 tissues, of which 223 were differentially expressed. Post-hoc analyses identified that 57 miRNAs were differentially expressed between the 3 tissues ($p<0.05$ in 3 combinations, i.e. BAT vs WAT, WAT vs muscle and muscle vs BAT). As shown in the heat map, an additional 19 miRNAs (framed in red) had a higher expression level in BAT in comparison to muscle and WAT. Post-hoc analyses also identified 166 miRNAs (table on the right of the heat map) that were specifically expressed in 1 or 2 tissues ($p<0.05$ in one or two of the combinations mentioned above). Another 35 miRNAs had higher expression levels in BAT vs muscle and WAT. So when adding all the significant highly expressed miRNAs found in BAT (6+4+19+35), a list of 64 BAT-enriched miRNAs was obtained.
