# Supplementary material for: Comparative analysis of microRNA expression in mouse and human brown adipose tissue
Source: BMC Genomics. 2015 Oct 19;16:820. doi: 10.1186/s12864-015-2045-8 (PMC4617708; doi:10.1186/s12864-015-2045-8)
Supplement: Additional file 4: — Table of the 145 miRNAs commonly expressed in both human and mouse BAT. (PDF 310 kb) [file 12864_2015_2045_MOESM4_ESM.pdf]

Additional file 4: Table of the 145 miRNAs commonly expressed in both human and mouse BAT.

**145 miRNAs commonly expressed in both human and mouse BAT**				
let-7a	miR-142-3p	miR-19b	miR-30d	miR-433
let-7b	miR-143	miR-200c	miR-30e-3p	miR-451
let-7c	miR-145	miR-203	miR-320	miR-455-5p
let-7d	miR-146a	miR-204	miR-323-3p	miR-455-3p
let-7e	miR-146b	miR-20a	miR-324-3p	miR-484
let-7f	miR-148a	miR-20b	miR-328	miR-486
let-7g	miR-149	miR-21	miR-331-3p	miR-489
miR-100	miR-150	miR-210	miR-335-5p	miR-491
miR-101a	miR-151-3p	miR-212	miR-337-5p	miR-494
miR-103	miR-151-5P	miR-214	miR-338-5P	miR-495
miR-106a	miR-152	miR-218	miR-339-3p	miR-500
miR-106b	miR-155	miR-22	miR-339-5p	miR-511
miR-10a	miR-15a	miR-22#	miR-340-5p	miR-532-3p
miR-10b	miR-15b	miR-222	miR-342-3p	miR-532-5p
miR-125a-5p	miR-16	miR-223	miR-345-5p	miR-574-3p
miR-125b-5p	miR-17	miR-224-5p	miR-34a	miR-598
miR-126-3p	miR-181a	miR-24	miR-365	miR-652
miR-127	miR-185	miR-25	miR-370	miR-664
miR-130a	miR-186	miR-26a	miR-376a	miR-7#
miR-130b	miR-18a	miR-26b	miR-376c	miR-708
miR-132	miR-191	miR-27a	miR-378	miR-720
miR-133a	miR-192	miR-28	miR-379	miR-744
miR-133b	miR-193	miR-29a	miR-382	miR-9
miR-134	miR-193b	miR-29c	miR-409-3p	miR-92a
miR-138	miR-194	miR-301a	miR-410	miR-93
miR-139-3p	miR-195	miR-30a	miR-411	miR-93#
miR-139-5p	miR-197	miR-30a-3p	miR-423-5p	miR-99a
miR-140	miR-199a-3p	miR-30b	miR-425	miR-99b
miR-140-3p	miR-19a	miR-30c	miR-431	miR-99b#
