# Supplementary material for: Comparative analysis of microRNA expression in mouse and human brown adipose tissue
Source: BMC Genomics. 2015 Oct 19;16:820. doi: 10.1186/s12864-015-2045-8 (PMC4617708; doi:10.1186/s12864-015-2045-8)
Supplement: Additional file 5: — Table of the 25 mouse BAT-enriched miRNAs in human BAT and their predicted gene targets involved in cellular growth, proliferation and differentiation. (PDF 429 kb) [file 12864_2015_2045_MOESM5_ESM.pdf]

Additional file 5: Table of the 25 mouse BAT-enriched miRNAs in human BAT and their predicted gene targets involved in cellular growth, proliferation and differentiation.

(1/5)

miRNA ID	Gene Symbol
let-7g	*ABL2, ACP1, ACTA1, ACVR1B, ACVR1C, ACVR2A, ACVR2B, ADCY9, ADRB1, ADRB2, ADRB3, AGO1, AGO3, AGO4, AKT2, APC2, ARHGEF7, BCL2L1, CALM1 (includes others), CAPN3, CASP3, CBL, CCND1, CCNF, CD86, CDC34, CDC42SE1, CDK6, CDKN1A, CHUK, CLDN12, CLDN16, CPSF4, CSNK1D, DOCK3, DPYSL3, DSP, DVL3, EEF2K, EIF2S2, EIF3J, EIF4G2, ELF4, EPB41, F2, FAM208A, FAS, FASLG, FGF11, FGF5, FRS2, FZD3, FZD4, GAB2, GHR, GLRX, GNAL, GNAT1, GNG5, GRIK2, GYS1, HAND1, HMOX1, HRAS, IGF1, IGF1R, IGF2BP1, IKBKAP, IKBKE, IL10, IL6, INSR, IRS2, ITGA11, ITGB3, ITGB8, KPNA1, KPNA4, KRAS, LIMK2, MAP3K1, MAP3K2, MAP3K3, MAPK11, MAPK9, MEF2C, MEF2D, MLLT4, MYC, NAPEPLD, NEDD4, NGF, NOS1, NRAS, NTRK3, NXN, OSMR, PAK1, PDGFB, PHC3, PLA2G3, PLD3, POLR2C, POLR2D, POLR3D, PPP2R2A, PRKAA2, PRKAB2, PTGS2, RAB8B, RAG1, RALB, RANBP2, Ras, RB1, RHOB, RHOG, RICTOR, RPL36A/RPL36A-HNRNPH2, RPS6KA3, SMAD2, SMARCAD1, SOCS4, SOCS7, TAB2, TGFBR1, TGFBR3, TP53, TSC1, TSPAN2, VIM, WASL, WNT1*
miR-126-3p	*CRK, CRKL, GNA13, IRS1, PIK3R2, VEGFA*
miR-132	*ACVR1, ACVR2B, ADCY1, ADCY3, AGO1, ARHGAP5, BMP3, BRCA1, CAPN8, CBLL1, CDC42BPA, CDKN1A, CFL2, CHRNA5, CPSF6, CREB5, CRK, CTGF, DIAPH1, DPYSL3, EIF2S3, EIF4A2, EP300, EPB41, FGF7, FGFR3, FOXA1, FOXO1, FOXO3, FRS2, GAB1, GNA12, GNB1, GRM3, GSK3B, ISL1, KDM5B, KITLG, KPNA1, MAP3K3, MAPK1, MAPK3, MAPKAP1, MAPT, MEF2A, MEIS1, MITF, MMP9, NFAT5, PAIP2, PAPOLA, PIK3R3, PPP2CB, PPP2R5C, PPP2R5E, PPP3CA, PRKD1, PTCH1, PXN, RAP2B, RASA1, RB1, RHOQ, RPL13A, SLC23A2, SLC2A1, SMAD2, SMAD5, SOD2, TCF7L1, TCF7L2, TJAP1, TJP1, TLN2, YWHAG*
miR-140	*ACACA, ADCY6, BCL2L1, BMP2, CAPN1, CDYL, CREB1, DNM3, DPYSL2, EIF4G2, FGF9, GIT1, GLI3, GNA13, GNG12, GYS1, IGF1R, IGF2BP1, IGFBP5, KAT2B, LIFR, LMO7, MAGI2, MAPK1, MEF2D, MYH9, NFAT5, PAX6, PDGFRA, PITX2, PPP1CC, PPP1R12A, PPP2R3A, RALA, RAP1B, RHOU, RPS6KA3, RPS6KA6, SLC2A1, SMAD3, SMURF1, SOCS7, SYNJ1, TGFBR1, VEGFA, WASF1, WNT1, YES1*
miR-142-3p	*ABL2, ACVR2A, ADCY9, AKT1S1, APC, ARHGEF12, BCL2L1, CD8A, CFL2, CHRNE, CLDN12, CRK, CTNND1, CTTN, EPN1, FGF9, FKBP1A, FOXO1, GAB1, GHR, GNAQ, GNB2, HGS, IL6ST, INPP5F, ITGAV, ITGB8, ITPR3, KAT2B, LIFR, MAP3K11, MYH10, MYH9, MYLK, NR2F6, PIK3CG, PIK3R6, PLCB1, PPP3CA, PPP3R1, RAC1, RHEB, RICTOR, ROCK2, S1PR3, SH3GLB1, SOCS6, TAB2, TGFB2, TGFBR1, TNFRSF13C, WASL, XIAP, XPO1, ZEB1*
miR-150	*ACVR1B, AKT3, APC, CAMK2G, CAPN6, CAST, CBL, CDKN1B, CNGB1, CSF3, EIF4B, EIF4E, ELK1, EP300, FOXD3, FZD4, GAB1, GRB7, GSK3B, HOXC9, IGF2BP1, IL7, IPO5, ITGAX, ITGB3, MAP2K4, MAP3K12, MAPK13, NOTCH3, NTRK2, PARVA, PDGFB, PIM1, PRKAR1A, PRKCA, PVRL2, SLC2A1, SLC2A4, SOCS7, SP1, TEK, TP53, UBTF, VEGFA, ZEB1*
miR-155	*ABL2, ACTA1, ACVR2A, ACVR2B, AGO4, AGTR1, APC, BDNF, CAB39, CBL, CCND1, CLDN1, CREB1, CSF2RB, CSNK1G2, CTNNB1, CYR61, EPAS1, ETS1, F2, FGF7, FGF9, FOS, FOXO3, FZD5, GNA13, GNAS, GSK3B, IKBKE, IL6ST, INPP5D, ITK, JARID2, KDM5B, KPNA1, KPNA4, KRAS, MAP3K10, MAP3K13, MAP3K14, MEF2A, MEIS1, MET, MYLK, NFAT5, PAK2, PHC3, PIK3R1, PRKAR1A, PRKCI, RAB5C, RAP1B, RELA, RHEB, RHOA, RHOQ, RICTOR, RPS6KA3, RPS6KA6, RPTOR, S1PR1, SKI, SMAD1, SMAD2, SMARCA4, SMARCAD1, SOCS1, SOCS6, SOS1, SOX10, SP1, SP3, TAB2, TCF4, TCF7L2, TGFBR2, TRAF3, TXNRD1, TYRP1, YWHAE, YWHAZ, ZIC3*

Additional file 5: (2/5)

miRNA ID	Gene Symbol
miR-15a	*ABL2, ACACA, ACTR2, ACVR2A, ACVR2B, AGO1, AGO4, AK4, AKT3, ARHGAP26, ARHGAP5, ATG13, BAIAP2, BCL2, BDNF, BMI1, BMPR1A, CAB39, CALM1 (includes others), CAPN6, CCND1, CCNE1, CD80, CDC42, CDC42EP2, CDK6, CFL2, CHP1, CHRNE, CLDN12, CLDN2, CREB5, CRKL, DIAPH1, DLL1, DRD1, EGFR, EIF2B5, EIF3A, EIF4B, EIF4E, EIF4G2, EXOC5, F2, FASN, FERMT2, FGF1, FGF18, FGF2, FGF7, FGF9, FGFR1, FIGF, FKBP1A, FOXO1, FRS2, FZD10, FZD4, FZD6, GHR, GIT1, GLRX, GNA12, GNA13, GNAI3, GNAT1, GRB10, GRIN1, GRM7, HMOX1, HSP90B1, HTR2A, HTR2C, IGF1, IGF1R, IKBKB, IL15, INSR, IRS1, IRS2, ITGA10, ITGA2, JARID2, JUN, KDR, KPNA1, KPNA3, KPNA4, KRAS, LIMS1, LIPE, MAP2K1, MAP2K4, MAP3K13, MAP3K4, MAP3K9, MAPK9, MAPRE1, MCL1, MKNK1, MRAS, MYLK, MYLK3, NAPEPLD, NFATC3, NOS1, NOTCH2, NRP2, OCRL, OTX1, PAK2, PAK7, PFKFB4, PHC3, PIK3C2A, PIK3R1, PIM1, PLCD1, PLXNB1, PPAP2B, PPM1A, PPM1D, PPP2R1A, PPP2R5C, PRKAB2, PRKAR2A, PRKCD, PRKG1, PTCH1, PTCH2, PTGS2, PTPRM, PVRL1, PVRL2, RAB8B, RAD9A, RAF1, RAPGEF1, RHOT1, RICTOR, RIF1, RPS6KA3, RPS6KA6, SALL4, SEMA3A, SH2D2A, SKI, SKIL, SLC2A3, SMAD3, SMAD5, SMAD7, SMURF1, SMURF2, SOCS6, SOS1, SOS2, SPTBN2, SYNJ1, TBP, TCF3, TGFBR3, TNFSF13B, TNPO1, TRAF3, TRAF6, TSC1, TSPAN5, TUBA1A, TXNRD3, VAV2, VEGFA, WASL, WIPF1, WNT3A, WNT4, WNT5B, WNT7A, YWHAH, YWHAQ, ZAK, ZFHX3, ZNF423, ZYX*
miR-18a	*ATM, CDK2, CDKN1A, CTGF, E2F1, FGF1, FGFR3, FNBP1, FOXH1, FRS2, HIF1A, IGF1, INPPL1, KDM5B, KIT, KPNA6, LIF, MAP3K1, MEF2D, MYLK, NEDD4, NEDD9, NFAT5, NOS1, NOTCH2, OCRL, OTX1, PHC3, PIAS3, RAB5A, RAB5C, RUNX1, SIX3, SMAD2, YBX3, YWHAB, ZAK*
miR-193	*ADCY9, ARHGEF12, ARNT, ATM, CALM1 (includes others), CCND1, CHRNB1, DVL3, EIF2AK1, EIF4B, ETS1, FGF1, GNAO1, GRB7, HOXC9, IGF2BP1, IGFBP5, ITPR2, KIT, KRAS, LIMS1, MAP3K3, MAPK10, MCL1, NFKBIE, OSMR, PLAU, POLR2J2/POLR2J3, PPP2R5C, PTK2, SF3A1, SLC23A2, SOS2, TCF4, TGFB2, TGFBR3, TSC1, UBA52, YWHAZ*
miR-19a	*ACTN1, ADCY1, ADCY7, ADCY9, ADRB1, AGO1, AK3, AK4, ARHGAP1, ARHGAP5, ARHGEF12, BCAR3, BMP3, BMPR2, CAB39, CALM1 (includes others), CAST, CCND1, CDC42BPA, CGN, CLIP1, CNGA3, CNR1, CNTFR, CREB5, CRMP1, CSF2RB, CSNK1G1, CTGF, DBN1, DOCK3, DPYSL5, EIF4A2, EIF4G2, ELAVL1, ELK3, EPN2, EXOC5, FGF6, FLNC, FRS2, FZD3, FZD6, GIT2, GRB10, GRIN2A, GSC, GSK3B, ID2, IGF1, IGFBP3, IL6ST, INHBB, ITGA11, ITGA2, ITGA6, ITGB3, ITGB8, JARID2, KIT, KPNA3, KPNA4, KPNA6, KRAS, LIF, MAGI2, MAP2K3, MAP3K12, MAP3K14, MAP3K2, MAPK1, MAPK14, MEF2A, MEF2C, MEF2D, MPRIP, NEUROG1, NRP2, OCRL, PAK6, PAX6, PFKFB3, PIK3C2A, PIK3CA, PIK3R3, PLA2G10, PLCL1, PLCL2, PPM1A, PPP1R12A, PPP2R4, PPP2R5A, PPP2R5E, PRKAA1, PRKAA2, PRKACB, PRR5L, PTEN, PTK2B, RAB5B, RAB8B, RAF1, RAP1A, RAP1B, RAP2B, REST, RFX4, RHOB, RICTOR, RPS6KA2, RPS6KA5, RUNX3, S1PR1, S1PR2, SLC2A1, SMAD4, SMAD5, SMARCA2, SMARCD2, SMURF1, SOCS1, SOCS3, SOCS5, SOCS6, SPHK2, STAT2, TGFBR2, TGIF1, TLN2, TNF, TNIP1, TSC1, VAV2, WNT1, WNT10A, WNT3, WNT7B, XIAP, YES1, ZAK, ZFYVE9*

Additional file 5: (3/5)

miRNA ID	Gene Symbol
miR-203	*ABL1, ACVR2A, ACVR2B, ADCY9, AK4, AKT2, ANGPTL1, APC, ARHGAP1, ATM, BIRC5, BMI1, CAB39, CAV1, CBL, CLDN1, CNTFR, CREB1, CREB3, CRK, CUL1, DGKZ, DNM1L, EIF4E, ETS2, FAM208A, FGF2, FGF5, GLI3, GNG2, GNG4, GYS1, HLA-DOA, HTR2A, HTR2C, ID4, IGFBP5, IL15, IRS2, ITGA2, ITGAV, ITPR2, KAT6A, KPNB1, KRT1, LIFR, MAGI1, MAP3K1, MAP3K13, MAP3K2, MAPK10, MAPK9, MAPRE1, MEF2C, MLLT4, NEDD9, NR5A2, NUDT21, OCLN, PDGFD, PDGFRA, PHC3, PIK3CA, PIK3CD, PLD1, PLD2, PPAP2B, PPM1A, PPM1D, PPP1CB, PPP1R12A, PRKAB1, PRKAB2, PRKAG2, PRKCA, PRKCB, PRKCI, PTCH1, PTPN1, PXN, RALB, RAP1A, RAP2A, RAPGEF1, RHOQ, RPL23A, RUNX2, SEMA3A, SIX3, SLC2A3, SMAD3, SMAD9, SMURF1, SNAI2, SOCS3, SOCS6, SOCS7, SP1, SP4, SPTBN1, SRC, SYNJ1, TCF12, TCF4, TESK1, TGFB2, TNPO1, VEGFA, YWHAQ, ZAK, ZEB1, ZFHX3*
miR-20a	*ABL2, ACVR1B, ADRA1B, AGO1, AK4, AKT3, ARHGAP1, ARHGAP26, ARHGEF7, BCL2, BMP2, BMPR2, CASP7, CCND1, CDK6, CDKN1A, CFL2, CNGB3, CREB1, CREB5, CRK, CSNK1G1, DNM2, DPYSL2, DPYSL5, DRD1, E2F1, EIF4G2, ELK3, EPB41, F2R, FGD1, FGF12, FGF4, FGF5, FLT1, FNBP1L, FOXA1, FRS2, FZD3, FZD4, FZD7, GAB1, GIT2, GNB5, GUCY1A3, HIF1A, IGF2BP1, IL6ST, IL8, ITGA4, ITGB8, ITPR2, JAK1, KAT2B, KPNA2, KPNA3, KPNA4, LIF, LIMK1, MAP3K1, MAP3K11, MAP3K12, MAP3K13, MAP3K14, MAP3K2, MAP3K3, MAP3K5, MAP3K8, MAP3K9, MAPK1, MAPK9, MAPRE1, MAPRE3, MCL1, MEF2D, MTMR2, MYF5, NAPEPLD, NEUROG1, NFAT5, NRP2, NTRK3, OCRL, OSM, PAK7, PAPOLA, PDGFRA, PFKFB3, PFKP, PHC3, PIK3R1, PLA2G6, PLCB1, PLS1, PLXNA1, PPARG, PPM1A, PPP2CA, PPP2R2A, PPP2R3A, PPP3R1, PRKACB, PTEN, RAB5B, RAB8B, RAPGEF1, RB1, REST, RHOC, RND3, RPS6KA1, RPS6KA2, RPS6KA3, RPS6KA4, RPS6KA5, RPS6KA6, RUNX1, RUNX3, S1PR1, SEMA7A, SKI, SLC2A4, SMAD4, SMAD5, SMAD6, SMAD7, SMURF1, SOCS6, SOCS7, SOS1, SSX2IP, STAT3, STK11, TCF4, TCF7L1, TGFB1I1, TGFBR2, TNFSF11, TNS1, ULK1, VASP, VAV2, VCL, VEGFA, VIM, XIAP, YES1, ZFYVE9*
miR-25	*ABL2, ACTC1, ADCY3, ADRB1, AGGF1, AGO3, ARF1, BMP7, BMPR2, CALM1 (includes others), CAMK2A, CCNE2, CDC42, CDC42BPA, CDC42EP2, CDK6, CDKN1A, CEBPA, CFL2, CLDN11, CNR1, CREB1, DDIT4, EIF4G2, EOMES, EXOC5, FKBP1A, FRS2, FZD10, GATA6, GIT2, GRIA1, GRIA3, GRIA4, GRM2, HAND1, HNF1B, IL6ST, IQGAP2, IRS2, ITGA5, ITGA6, ITGAV, ITPR1, JARID2, KAT2B, MAP2K4, MAP3K13, MAP3K2, MCL1, MEF2D, MITF, MYH9, NFAT5, NOTCH1, PAIP1, PAX3, PFKFB4, PIK3R3, PPAP2B, PPP1R12A, PRKAB2, PRKAR1B, PRKAR2B, PRKCE, PTEN, PVRL1, RAB8B, RAG1, RAP1A, RAP1B, REST, RPL15, RPS6KA4, S1PR1, SEMA3A, SKI, SLC2A3, SMAD6, SMAD7, SMURF1, SNAI1, SOCS6, SP1, SPHK2, SYNJ1, TCF4, TGFB2, TGIF1, TNPO1, TOB1, TRAF3, TSC1, ULK1, VCL, WASL, WNT5A, WWP2, ZAK, ZFHX3*
miR-26b	*ABL2, ACVR1C, ACVR2B, AGO1, ARHGAP26, ARHGEF12, ATF2, CBLL1, CDH2, CDK6, CNR1, CPSF2, CREB1, CREBBP, CSNK1G1, CTGF, EIF2S1, EIF3A, EIF4G2, EIF5, FAM208A, FGD1, FGF9, GATA4, GNA13, GRB10, GSK3B, HGF, HNF1B, HOXC9, HTR2C, IGF1, INHBB, ITGA4, ITGA5, ITGA6, ITGB8, ITPR1, JARID2, KPNA2, KPNA3, KPNA6, LEF1, LIF, LIFR, LIMS1, MAP3K1, MAP3K2, MAP3K9, MCL1, MEF2C, MITF, MRAS, MYH10, MYLK3, NRAS, OCLN, PAK2, PFKFB2, PFKFB3, PIK3C2A, PIK3R3, PIM1, PLCB1, PLCL1, PPM1B, PPP2R3A, PPP2R5A, PPP3CB, PPP3R1, PRKAA2, PRKAG2, PRKCD, PRKCQ, PRR5L, PTEN, PTGS2, RAP1A, RB1, REST, RHOQ, RHOU, RPS6KA2, RPS6KA6, SLC2A3, SMAD1, SMAD2, SMAD4, SOCS6, SOCS7, SPTBN1, SSX2IP, TCF12, TCF7L2, TGFBR2, TNPO1, TOB1, TRAF3, TXNRD3, UBTF, ULK1, WDR33, WNT5A, WWP2, YWHAE, ZFHX3*

Additional file 5: (4/5)

miRNA ID	Gene Symbol
miR-30b	*ABL1, ABL2, ACTC1, ACTN1, ACVR1, ADRA1D, ADRA2A, ADRA2B, ADRB1, AGO1, AGO3, ARHGAP26, ARHGEF6, ARPC5, BCL2, BDNF, BMP7, CAMK2D, CAMK4, CAPN5, CCNE2, CFL2, CNGB3, CPSF6, CRKL, CSNK1A1, CSNK1G1, CTGF, DDIT4, DGKZ, DPYSL2, EPB41, EPN2, F2, FOXA1, FOXO3, FRS2, FZD2, FZD3, GDNF, GLI2, GNA13, GNAI2, GNAO1, GNG10, GRB10, GRIA2, GRIN2A, GRM3, GRM5, GUCY2C, IGF1, IGF1R, IL1A, IRS1, IRS2, ITGA2, ITGA4, ITGA5, ITGA6, ITGB3, JAK1, JARID2, JUN, KPNA3, KPNA6, KRAS, LIFR, LYN, MAGI2, MAP3K1, MAP3K12, MAP3K13, MAP3K2, MAP3K5, MEF2D, MET, MPL, MYH10, MYH11, NEDD4, NFAT5, NFATC3, NOTCH1, NR2F2, NR5A2, NRP2, PAPOLA, PAX3, PDGFRB, PHC3, PIK3CD, PIK3R2, PLCB4, PLCG1, PLS1, PLXNA1, PPP1R12A, PPP2R4, PPP3CA, PPP3CB, PPP3R1, PRKAR1A, RAB7A, RAP1B, RAP2B, RASA1, RHOB, RPS6KA2, RUNX1, RUNX2, SEMA3A, SERPINE1, SMAD1, SMAD2, SMARCD2, SNAI1, SNAI2, SOCS1, SOCS3, SOCS6, SOS1, SP4, SSX2IP, TCF7, THEM4, TIMP3, TNIP1, TNPO1, TP53, TRAF3, TSC1, TSPAN2, VAV2, WIPF1, WNT5A, WNT7B, XPO1, YWHAZ*
miR-320	*ACVR2B, AK4, AKT3, ANGPTL1, ARF1, ARHGAP26, ARHGAP5, ARPC5, ASH2L, BMI1, BMP3, BMP6, BMPR1A, CALM1 (includes others), CBL, CBLL1, CDH2, CDK6, CREB5, CRK, CRKL, CSNK1E, CSNK1G3, DBN1, DPYSL3, E2F1, EIF2B1, EIF4B, EPN2, EPO, ETS2, FAM208A, FGF9, FLNC, FOXO3, FZD3, GNAI1, GNAZ, GRB2, HLTF, ID4, IGF1, IGF1R, IPO5, ITGB3, KITLG, MAGI1, MAPK1, MAPK9, MCL1, MYH10, MYL12A, NFATC3, NRP1, ONECUT1, PAK7, PFKFB2, PFKM, PHC1, PIK3R1, PLCG1, PPP1CB, PPP2R2C, PPP2R5B, PRKAG2, PRKAR1A, PRKG1, PTEN, RAC1, RAD9A, RANBP2, RAP1A, RAP2B, RASA1, RPL15, RSU1, RUNX1, S1PR1, SEMA3A, SMAD4, SMARCD2, SMURF1, SP1, TGFBR1, TGFBR2, TNPO1, TSC1, TUBA8, ULK1, VIM, XIAP, XPO1, YES1, YWHAG, YWHAQ, YWHAZ, ZIC3*
miR-328	*ADCY1, ARPC4, CLDN19, CNR1, CPT1A, EIF4EBP1, ELF1, EPO, FGD1, FGF11, FOXA2, FZD3, FZD7, GLRX, IGF1R, IL5, IL8, ITGA5, MAGI1, MAP2K7, MAP3K9, MAPK14, NFATC4, PAK6, PHC3, PIM1, PLA2G4C, PLCE1, PNPLA2, PPP2R5D, SLC2A1, TCF7L2, TGFB2, TNS1, TSC1, TYR, WASF2, YWHAZ, ZAK*
miR-338-5P	*PHC3*
miR-365	*ACVR1, ACVR2B, ADCY6, AGO4, AKT3, BCL2, BDNF, CCND1, CHP1, CLDN11, CPT1A, CREB5, CSF2RB, CSK, CSTF1, EPAS1, ETS1, FGF1, FRS2, FZD3, FZD5, GNAT1, IL1A, ITGA10, MAP3K13, MAPK1, MEF2D, MEIS1, MTMR2, MYLK, PAX6, PIK3R3, PLCB4, PPM1D, PRKAR2A, PRKG1, RHEB, RICTOR, RPS6KA6, SEMA3A, SET, SKI, SMAD2, SOCS5, SOCS7, SOS1, SYNJ1, WNT5A, YWHAH*
miR-423-5p	*ACACA, ACVR1B, AK1, BAIAP2, BMP8B, CALM1 (includes others), CALML5, CAPN5, CCNF, CD8A, CDC42SE1, CDKN1A, CLDN19, CLDN23, CRHR2, CSF1, DOCK1, DPYSL4, DVL1, EIF4EBP1, ELF4, EPN2, EXOC3, FGFR3, GNAI2, GRIK3, GRIN1, GRM2, GYS1, INPPL1, ITGA5, KEAP1, LIMK1, MAP3K14, MEF2D, MRAS, NFATC1, NFATC2, NOS1, NPM2, PAK6, PDGFRB, PDPK1, PLA2G2F, PLA2G6, PLCB1, PRKACA, RAC2, RCC1, RPS6KA4, S1PR3, SGSM3, SHC1, TGFBR3, TSPAN3, TUBB6, UBA52, WNT3A*
miR-451	*CAB39, CAV1, MEF2D, MYLK3, NEDD9, NR5A2, S1PR2, TSC1, TTN, YWHAZ*
miR-455	*ARHGEF12, ARNT, BMP8A, BMPR2, CAMK4, CAST, CDKN1B, CTNND1, EPN2, ETS1, FZD5, IGF1R, INPP5K, ITGB8, KDR, KPNA1, KPNA3, MKNK1, NCK2, PARD3, PARVA, PLD1, PPP1R12A, PRKCI, RAF1, RUNX2, S1PR1, SOCS3, SYNJ2, TJP1, TNPO1, TSPAN5, TUBB*

Additional file 5: (5/5)

miRNA ID	Gene Symbol
miR-484	*CSF1, F2RL3, FGF1, FLNB, HNF1A, MAGI1, MAPKAPK2, PDGFA, PIK3CD, TGFBR3, WNT2B*
miR-491	*ARF3, BCL2L1, CAMK2B, CAPN5, CSNK1G1, EXOC8, FGD1, GATA4, GIT1, GNAT1, IGF2, IGF2BP1, INPP5F, IQGAP3, ITGAX, KDM5B, LIMK1, MAP3K11, MAPKAP1, MPL, MYL9, PARVA, PDGFRA, PHC3, PTPN11, PVRL1, PXN, RAB7A, RND2, RPS24, RPTOR, SF3A1, SLC2A1, SNAI1, SPTB, TCF7, TNS1, TXNRD1*
